# Molecular mechanisms by which tumor cell CD73-mediated adenosine signaling drives M2 polarization of macrophages in non-small cell lung cancer

**DOI:** 10.3389/fcell.2026.1875070

**Published:** 2026-08-14

**Authors:** Qi Zheng, Binbin Qin, Bin Shen, Yingjun Huang, Jiayan Zhu, Jingwei Hu, Xingdong Zheng, Jingyi Cai, Ying Xie, Yajun Song

**Affiliations:** Department of Respiratory and Critical Care Medicine, Shanghai Second People’s Hospital, Shanghai, China

**Keywords:** A2A receptor, adenosine pathway, CD73, macrophage M2 polarization, non-small cell lung cancer

## Abstract

**Background:**

Immune checkpoint inhibitors have improved outcomes for a subset of patients with non-small cell lung cancer (NSCLC), but the immunosuppressive tumor microenvironment (TME) limits their efficacy. Polarization of tumor-associated macrophages (TAMs) toward an M2 phenotype is a key feature of immune escape in NSCLC.

**Objective:**

Using the CD73 (NT5E)-mediated adenosine metabolic pathway as an entry point, to elucidate its role and molecular mechanisms in driving immunosuppressive TAM polarization in NSCLC.

**Methods:**

Using TCGA datasets and clinical tissue samples, we analyzed correlations between CD73 expression, macrophage infiltration, and patient prognosis. NSCLC cell lines were used to establish CD73 knockdown/overexpression and pharmacological inhibition models and to quantify extracellular adenosine generation. Tumor cell-conditioned media and Transwell co-culture systems were constructed to induce THP-1 macrophage polarization. Flow cytometry, qPCR, and ELISA were used to assess M1/M2 markers and cytokine profiles. Rescue experiments were performed by supplementing exogenous adenosine or 5’-N-ethylcarboxamidoadenosine (NECA), and adenosine dependence was validated using an A2A receptor antagonist together with downstream cyclic adenosine monophosphate (cAMP)-protein kinase A (PKA)-cAMP response element-binding protein (CREB) signaling measurements.

**Results:**

TCGA-based and clinical-sample analyses suggested that high CD73 expression was associated with poorer prognosis and increased infiltration of M2-like macrophages. *In vitro* experiments confirmed that CD73 expression in NSCLC cells correlated positively with extracellular adenosine production. Pharmacological inhibition or knockdown of CD73 significantly reduced adenosine levels and weakened tumor-derived induction of macrophage M2 polarization, manifested as downregulation of M2 markers with enhancement of the M1 phenotype. Exogenous adenosine supplementation restored M2 polarization, whereas blockade of the A2A receptor or inhibition of the PKA/CREB pathway reversed this polarization effect and attenuated downstream CD8^+^ T-cell dysfunction-associated changes.

**Conclusions:**

CD73-driven adenosine signaling promotes immunosuppressive M2 polarization of NSCLC-associated macrophages through the adenosine-A2A receptor (A2AR)-cAMP/PKA/CREB signaling axis, providing *in vitro* mechanistic evidence supporting CD73 targeting to improve the tumor immune microenvironment.

## Introduction

Non-small cell lung cancer (Non-Small Cell Lung Cancer, NSCLC) accounts for 80%–85% of all lung cancer cases and remains the leading cause of cancer-related death worldwide (Bray et al., 2018). Over the past decade, immune checkpoint inhibitor (ICI) therapies targeting programmed cell death protein-1 (PD-1) and its ligand (PD-L1) or cytotoxic T-lymphocyte-associated protein 4 (CTLA-4) have profoundly reshaped the clinical treatment landscape for NSCLC (Luo et al., 2021). In particular, for unresectable stage III NSCLC, the PACIFIC trial established durvalumab (an anti-PD-L1 monoclonal antibody) as the global standard of care for up to 12 months of consolidation therapy after concurrent chemoradiotherapy (cCRT) (Antonia et al., 2017). Long-term follow-up showed that the 5-year progression-free survival (PFS) and overall survival (OS) rates in the durvalumab group reached 33.1% and 42.9%, respectively, significantly outperforming placebo (Spigel et al., 2022). However, despite these breakthroughs, more than half of patients exhibit primary or acquired resistance to ICIs, leaving overall response rates at a relatively limited plateau (Sharma et al., 2017).

One of the central drivers of this resistance is a highly complex, immunosuppressive tumor microenvironment (Tumor Microenvironment, TME) (Binnewies et al., 2018). Within the TME, tumor cells recruit and “educate” host immune cells and stromal cells through multiple mechanisms, collectively building a robust metabolic and molecular barrier that enables evasion of immune surveillance (Hanahan and Weinberg, 2011). Among the diverse cellular populations that constitute an immunosuppressive TME, tumor-associated macrophages (Tumor-Associated Macrophages, TAMs) play a pivotal role and often account for a substantial proportion of tumor-infiltrating immune cells (Mantovani et al., 2017). Macrophages exhibit marked phenotypic plasticity and can polarize toward different functional states in response to cytokines and metabolic signals in the microenvironment: classically activated M1 macrophages are pro-inflammatory, anti-tumorigenic, and highly capable of antigen presentation, in contrast, alternatively activated M2 macrophages express high levels of scavenger receptor markers such as CD206 (MRC1), CD163, and CD204, and secrete immunosuppressive and pro-angiogenic factors such as IL-10 and vascular endothelial growth factor (VEGF) (DeNardo and Ruffell, 2019). In many solid tumors, including NSCLC, abundant infiltration of M2-like TAMs is not only closely associated with poor prognosis but also serves as a major driver of functional exhaustion of cytotoxic CD8^+^ T cells (Cassetta et al., 2019).

In recent years, purinergic signaling has emerged as a central hub linking tumor metabolic reprogramming and immune regulation. Under pathological stresses such as hypoxia, nutrient deprivation, and therapy-induced necrosis, tumor and stromal cells release large amounts of extracellular adenosine triphosphate (ATP) (Di Virgilio et al., 2018). Extracellular ATP initially functions as a damage-associated molecular pattern (DAMP) that can activate immunity and promote inflammation, however, in the TME, a highly expressed ectonucleotidase network-particularly CD39 (ENTPD1) and CD73 (NT5E)-rapidly dephosphorylates pro-inflammatory ATP stepwise and ultimately converts it into adenosine (Adenosine, Ado), a potent immunosuppressive metabolite (Allard et al., 2017). As the rate-limiting enzyme catalyzing the conversion of adenosine monophosphate (AMP) to adenosine, aberrant overexpression of CD73 in multiple malignancies directly leads to pathological accumulation of adenosine in the microenvironment (Antonioli et al., 2013). Free extracellular adenosine exerts broad biological effects mainly by binding four G-protein-coupled receptors on target cells (A1R, A2AR, A2BR, A3R) (Fredholm et al., 2001). Among them, the A2A receptor (A2AR), due to its high expression on key immune cell types (T cells, macrophages, dendritic cells) and high affinity for adenosine, has been recognized as a master receptor that mediates tumor immune escape (Ohta et al., 2006). When adenosine binds and activates A2AR on macrophages, the receptor-coupled Gs protein stimulates adenylyl cyclase (AC), causing a sharp rise in intracellular cyclic adenosine monophosphate (cAMP). cAMP accumulation further activates protein kinase A (PKA), activated PKA then enters the nucleus and phosphorylates the transcription factor CREB (cAMP response element-binding protein), thereby initiating transcriptional programs of anti-inflammatory and immunosuppressive genes (Wen et al., 2010).

Although adenosine signaling has been implicated in immune suppression, the specific sequence by which tumor-cell CD73-derived adenosine programs macrophages toward an M2-like phenotype in NSCLC remains insufficiently defined. In this study, we focused on the CD73-adenosine-A2A receptor (A2AR) axis and examined whether activation of cyclic adenosine monophosphate (cAMP)-protein kinase A (PKA)-cAMP response element-binding protein (CREB) signaling mediates macrophage reprogramming and subsequent suppression of CD8^+^ T-cell function. This streamlined experimental framework was designed to connect tumor metabolic remodeling with macrophage polarization and T-cell dysfunction without overextending the conclusions beyond the *in vitro* evidence.

## Materials and methods

### Reagents and materials

Adenosine monophosphate (AMP), adenosine, and the stable non-selective adenosine receptor agonist 5′-N-ethylcarboxamidoadenosine (NECA) were purchased from Sigma-Aldrich. Highly selective small-molecule inhibitors used for pharmacological interventions included the A2A receptor (A2AR) antagonist ZM241385, the A1 receptor antagonist DPCPX, the protein kinase A (PKA) inhibitor H89, and the selective cAMP response element-binding protein (CREB) inhibitor 666-15 (Xie et al., 2015). Specific antibodies used for flow cytometry and immunoblotting included antibodies against CD73, GAPDH, CD206, CD86, phosphorylated PKA substrates, phosphorylated CREB (Ser133), and total CREB. ELISA kits for quantifying IL-10, TGF-β, TNF-α, and cyclic adenosine monophosphate (cAMP) were purchased from R&D Systems.

### Clinical data acquisition and bioinformatics analysis

Transcriptomic and clinical follow-up data of NSCLC patients were obtained from public TCGA lung cancer cohorts (Cancer Genome Atlas Research Network, 2014). Kaplan-Meier analysis was used to compare overall survival (OS) between groups stratified by CD73 (NT5E) expression, with a follow-up window of 60 months. In addition, gene expression matrices from clinical samples were extracted, and Pearson correlation coefficients were computed to assess the linear correlation between NT5E expression and markers of alternatively activated (M2) macrophages, including CD206/MRC1, in tumor tissues.

### Ethics statement

The use of human tissue samples and healthy-donor peripheral blood samples was approved by the Ethics Committee of Shanghai Second People’s Hospital (Approval No.: [please insert the official ethics approval number]). Written informed consent was obtained from all participants before sample collection. The TCGA data used in this study were publicly available and de-identified; therefore, no additional institutional approval was required for the database analysis.

### Cell lines and primary cell culture

Multiple representative NSCLC-related cell lines were used for baseline screening, including the human NSCLC cell lines A549, PC9, H1975, and H1299, as well as the murine Lewis lung carcinoma (LLC) cell line. The LLC cell line was introduced only as a murine lung-cancer reference during the initial CD73 expression screen, because CD73 biology is frequently investigated in murine lung-cancer models. To avoid cross-species confounding, LLC was not included in any downstream experiments involving human THP-1-derived macrophages, primary human CD8^+^ T cells, conditioned media, rescue assays, receptor blockade, or signaling validation. A macrophage model was generated by PMA-induced differentiation of the human monocytic leukemia cell line THP-1 (Genin et al., 2015). Accordingly, all co-culture and conditioned-medium experiments involving THP-1 macrophages were performed exclusively with human NSCLC cell lines. Primary CD8^+^ T cells were isolated from peripheral blood mononuclear cells (PBMCs) of healthy donors, activated *in vitro*, and used for downstream co-incubation and functional validation assays.

### Construction of stable cell lines

After profiling baseline CD73 mRNA and protein expression across the cell lines by qPCR and Western blot, the H1299 cell line (highest endogenous expression) and A549 cell line (minimal expression) were selected as parental lines. Using a lentiviral vector system, shRNA targeting CD73 was introduced into H1299 cells to generate a stable knockdown line (H1299-shCD73), with a negative control group (shCtrl). A CD73 overexpression vector was introduced into A549 cells to generate a stable overexpression line (A549-CD73-OE), with an empty-vector control (Vector).

### *In vitro* co-culture models and pharmacological interventions

To assess paracrine effects of tumor-derived metabolites on myeloid cells, a non-contact Transwell co-culture model was established using human cell components only. Human NSCLC cells with different CD73 states (A549-CD73-OE or H1299-shCD73) were seeded in the upper chamber, and PMA-differentiated THP-1 macrophages were seeded in the lower chamber. The murine LLC line was excluded from all Transwell co-culture, macrophage polarization, T-cell functional, rescue, and signaling-blockade assays, thereby avoiding human-mouse cross-species co-culture bias. In mechanistic experiments, AMP substrate was supplemented to selected co-cultures, or adenosine/NECA was added at 10 μM and 50 µM for phenotypic rescue. In receptor and pathway blockade experiments, ZM241385, DPCPX, H89, or 666–15 was added in advance to treat cells.

### Macrophage phenotype assessment and flow cytometry

After co-culture, macrophage morphology was examined by light microscopy and the elongation index (length-to-width ratio) was calculated to distinguish the round morphology of unpolarized cells from an M2-like spindle-shaped phenotype. Macrophages from the lower chamber were collected, prepared as single-cell suspensions, stained with fluorophore-conjugated antibodies, and analyzed by flow cytometry to quantify the proportion of cells positive for the M2 marker CD206 and the M1 marker CD86.

### Quantification of metabolites, intracellular second messenger, and cytokines

After AMP substrate supplementation to tumor cell cultures, supernatants were collected and extracellular adenosine concentrations were quantified by high-performance liquid chromatography (HPLC). Supernatants from co-culture systems were also collected to quantify IL-10, TGF-β, and TNF-α concentrations using ELISA kits according to the manufacturers’ protocols. To quantify intracellular second messenger changes, macrophages were lysed and intracellular cAMP concentrations were measured by ELISA.

### Real-time quantitative PCR

Total RNA was extracted and reverse-transcribed into cDNA. qPCR was used to quantify the relative expression of the four adenosine receptors in macrophages (with a focus on A2A receptor/ADORA2A) and key downstream polarization genes (e.g., Arg-1).

### Immunoblotting analysis

Total protein or nuclear extracts were collected, separated by SDS-PAGE, and analyzed by Western blotting. Knockdown/overexpression efficiency of CD73 was quantified, PKA activity was assessed by measuring phosphorylated substrates, and the relative ratio of nuclear p-CREB (Ser133) to total CREB in macrophages was semi-quantified.

### Sequential T-cell co-culture and functional validation

To validate secondary immunosuppressive effects of polarized macrophages on T cells, a sequential co-culture system was established. Macrophages conditioned by different tumor cell-conditioned media were co-incubated with activated primary CD8^+^ T cells at a 1:5 ratio. T-cell proliferation was quantified, and flow cytometry was used to measure the expression of exhaustion markers TIM-3 and LAG-3. Subsequently, treated T cells were co-cultured with tumor target cells for functional cytotoxicity assays to quantify tumor-specific killing.

### Chromatin immunoprecipitation (ChIP)

To validate direct binding of p-CREB to target gene promoters, chromatin immunoprecipitation followed by quantitative PCR (ChIP-qPCR) was performed. After crosslinking and sonication, chromatin was immunoprecipitated using an anti-p-CREB antibody, and qPCR was used to quantify enrichment of DNA regions within the IL-10 promoter.

### Dual-luciferase reporter assay

To assess transcriptional activation capacity, luciferase reporter plasmids containing the wild-type IL-10 promoter or a mutant promoter with a disrupted CREB-binding motif were constructed. Following transfection and stimulation with different conditioned media, relative luciferase activity (RLU) was quantified using a dual-luciferase reporter system.

### Statistical analysis

Data are presented as mean ± standard deviation unless otherwise indicated. Comparisons between two groups were performed using Student’s t-test, and comparisons among multiple groups were performed using one-way analysis of variance followed by an appropriate *post hoc* test. Survival curves were analyzed using the Kaplan-Meier method and log-rank test. Correlations between gene-expression variables were evaluated using Pearson correlation analysis. A two-sided P value <0.05 was considered statistically significant.

## Results

### CD73 is highly expressed in NSCLC and is associated with poorer prognosis and M2 macrophage infiltration

**FIGURE 1:**
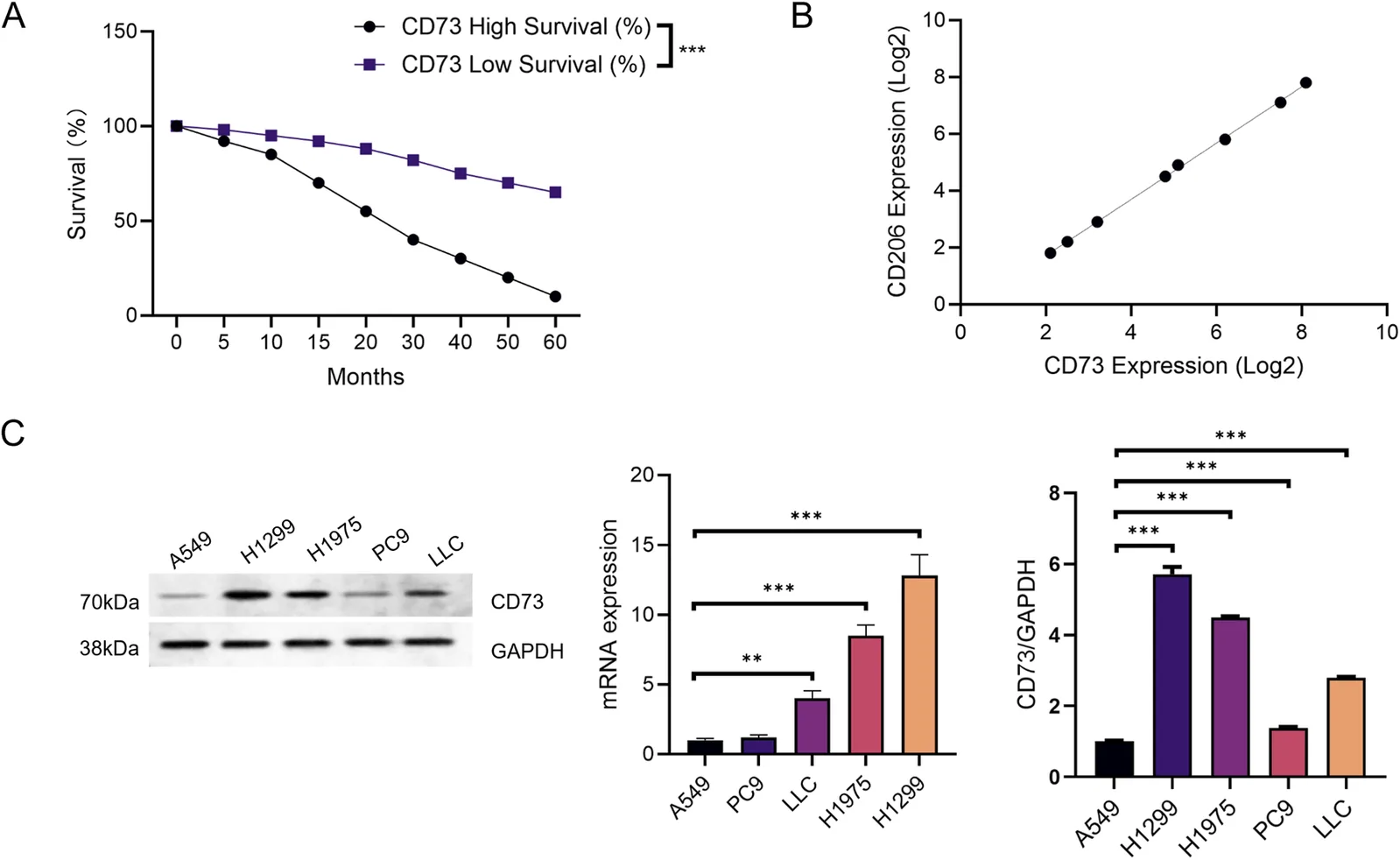
Clinical significance of CD73 in NSCLC and baseline CD73 expression in different cell lines. **(A)** Kaplan-Meier survival analysis based on TCGA lung cancer data comparing overall survival (OS) between NSCLC patients with high vs. low CD73 expression. **(B)** Pearson correlation analysis of CD73 (NT5E) expression and the M2 macrophage marker CD206 in clinical tumor samples. **(C)** Baseline CD73 mRNA and protein levels across five representative NSCLC-related cell lines (human A549, PC9, H1975, H1299, and murine LLC) measured by qPCR and Western blotting; LLC was included only for baseline expression screening and was not used in downstream human macrophage co-culture assays. Statistical significance is indicated by asterisks above the compared groups (*P < 0.05, **P < 0.01, ***P < 0.001).

To evaluate the clinical significance of CD73 (NT5E) in NSCLC, we first performed survival analysis using TCGA lung cancer data (Figure 1A). Patients were stratified into high- and low-CD73 groups according to CD73 (NT5E) expression. The high-CD73 group showed shorter overall survival than the low-CD73 group over the plotted 60-month follow-up window. Linear correlation analysis of gene expression in clinical samples (Figure 1B) further suggested a positive association between NT5E expression and M2 macrophage markers such as CD206/MRC1 in tumor tissues.

To establish subsequent *in vitro* models, baseline CD73 expression was profiled across representative NSCLC-related cell lines (human A549, PC9, H1975, H1299, and murine LLC) by qPCR and Western blotting (Figure 1C). LLC was included solely as a murine reference for the initial expression screen and was excluded from all downstream human macrophage co-culture and T-cell assays. Because the macrophage model used in downstream experiments was human THP-1-derived, only the human NSCLC cell lines were considered for subsequent co-culture experiments. CD73 expression was highest in H1299 cells (relative mRNA level up to 12.8, relative band density 3.8), whereas A549 cells showed minimal baseline expression (relative mRNA level 1.0, band density 0.2). Accordingly, H1299 and A549 were selected as parental lines for CD73 knockdown (KD) and overexpression (OE), respectively.

### Tumor cell-derived CD73 promotes adenosine accumulation and induces macrophage M2 polarization

**FIGURE 2:**
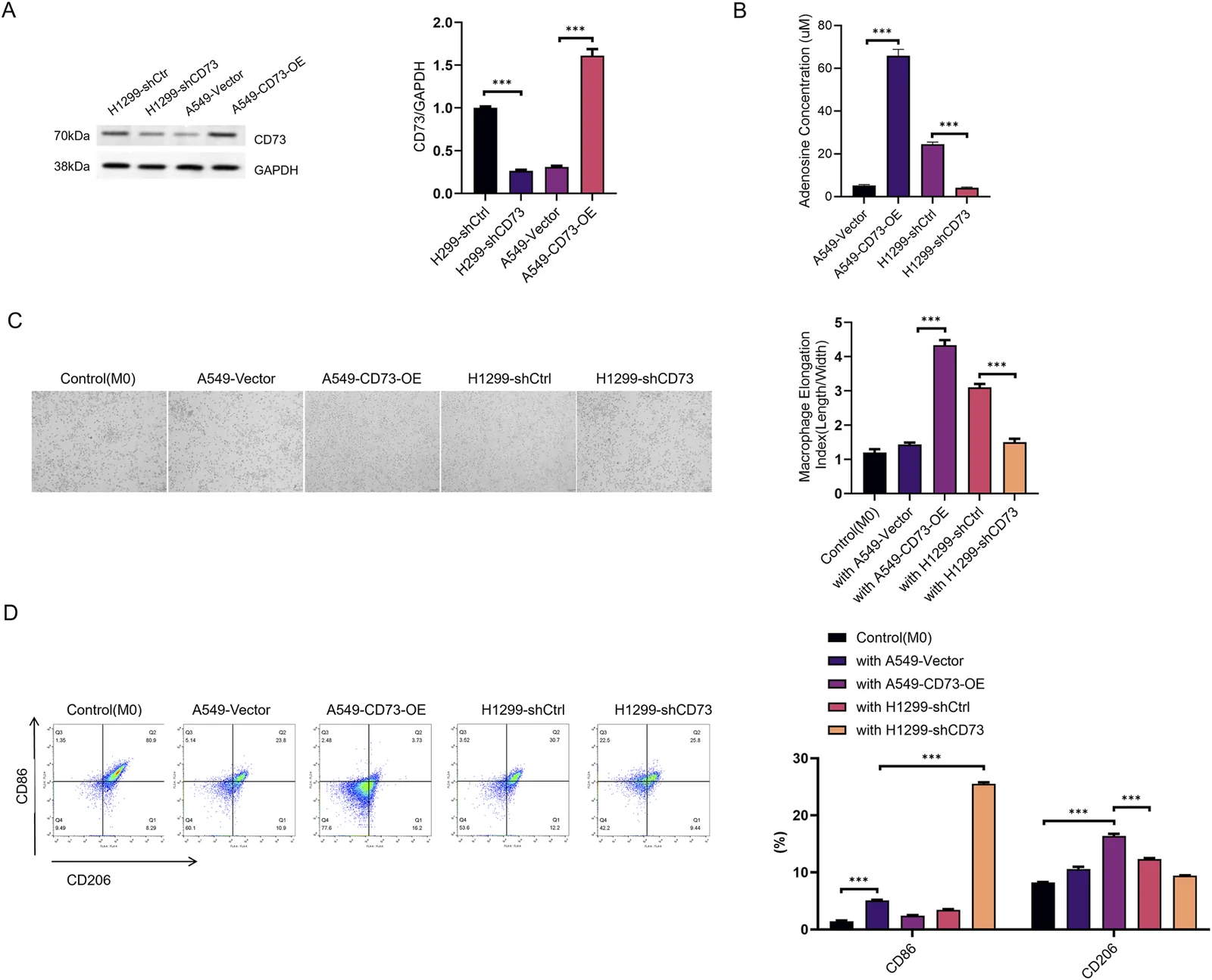
Effects of tumor cell CD73 expression on extracellular adenosine generation and macrophage polarization. **(A)** Western blot validation of stable CD73 knockdown (shCD73) in H1299 cells and stable CD73 overexpression (CD73-OE) in A549 cells. **(B)** HPLC quantification of extracellular adenosine generated after AMP supplementation in tumor cells with different CD73 states. **(C)** Light microscopy images of macrophage morphology after co-culture with tumor cells, with quantification of the elongation index. **(D)** Flow cytometry quantification of the proportions of macrophages positive for the M1 marker CD86 and the M2 marker CD206. Statistical significance is indicated by asterisks above the compared groups (*P < 0.05, **P < 0.01, ***P < 0.001).

Stable CD73 knockdown (H1299-shCD73) and CD73 overexpression (A549-CD73-OE) lines were constructed. Western blot quantification confirmed (Figure 2A) that CD73 protein levels in H1299-shCD73 were reduced to 0.15-fold of the shCtrl control, whereas CD73 levels in A549-CD73-OE increased to 42-fold of the vector control (from 0.1 to 4.2). HPLC quantification of culture supernatants showed that CD73 expression directly determined the capacity for extracellular adenosine generation (Figure 2B). After AMP supplementation, the A549-CD73-OE group produced adenosine concentrations as high as 65.8 µM, far exceeding the control (5.2 µM). Conversely, adenosine generation in H1299-shCD73 dropped significantly from 24.5 µM in controls to 4.1 µM.

Using a Transwell co-culture system, tumor cells with different CD73 states were co-cultured with THP-1-derived macrophages, resulting in marked morphological changes in macrophages (Figure 2C). Macrophages co-cultured with A549-CD73-OE cells became elongated with pseudopodia and adopted a typical spindle-shaped M2-like morphology, with an elongation index increasing to 4.5. In contrast, macrophages co-cultured with H1299-shCD73 cells largely retained a round morphology (elongation index reduced to 1.5), consistent with an M0/M1-like state. Flow cytometry analysis (Figure 2D) corroborated this phenotypic remodeling: in the CD73 overexpression-driven co-culture, the M2 marker CD206 increased to 75% positivity, whereas the M1 marker CD86 was strongly suppressed (6.5%). In the CD73 knockdown-driven H1299 co-culture, CD206 decreased to 12% and CD86 was rescued to 28%.

### CD73-mediated macrophage M2 polarization depends on adenosine-A2A receptor signaling

**FIGURE 3:**
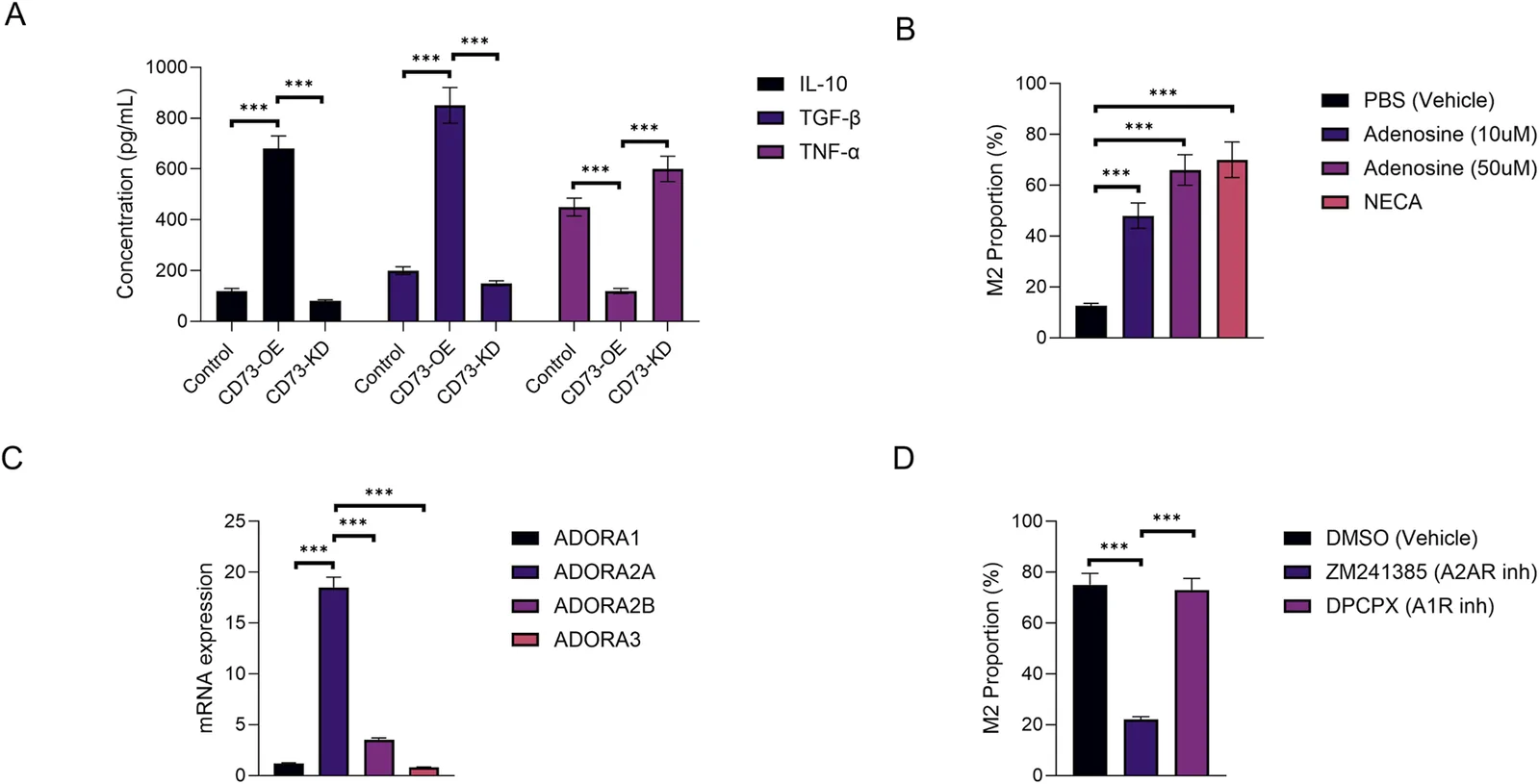
Macrophage M2 polarization depends on adenosine-A2A receptor signaling. **(A)** ELISA quantification of IL-10, TGF-β, and TNF-α in co-culture supernatants. **(B)** Rescue of M2 macrophage proportion (CD206+) by supplementing adenosine (10 μM, 50 µM) or 5′-N-ethylcarboxamidoadenosine (NECA) in the CD73 knockdown co-culture system. **(C)** qPCR profiling of macrophage adenosine receptors (ADORA1, ADORA2A, ADORA2B, ADORA3). **(D)** Effects of A2AR antagonist (ZM241385) and A1R antagonist (DPCPX) on M2 polarization in the CD73 overexpression co-culture system. Statistical significance is indicated by asterisks above the compared groups (*P < 0.05, **P < 0.01, ***P < 0.001).

To characterize the secreted cytokine profile underlying this phenotypic shift, ELISA was used to quantify cytokines in co-culture supernatants (Figure 3A). In the CD73-OE group, M2-associated cytokines IL-10 and TGF-β were specifically elevated (up to 680 pg/mL and 850 pg/mL, respectively), while the M1 cytokine TNF-α was suppressed to 120 pg/mL. In contrast, the CD73-KD group showed the opposite pattern (IL-10 decreased to 80 pg/mL, while TNF-α increased to 600 pg/mL).

To confirm adenosine as the key effector molecule in this polarization pathway, rescue experiments were performed (Figure 3B). In the H1299-shCD73 co-culture system where endogenous adenosine generation was impaired, the baseline M2 polarization rate (CD206+) remained at 12.5%. However, supplementation with 10 µM or 50 µM adenosine restored the M2 fraction in a dose-dependent manner to 48% and 66%, respectively. The stable adenosine receptor agonist NECA similarly rescued M2 polarization to 70%. Next, qPCR profiling of the four adenosine receptors in macrophages (Figure 3C) revealed that A2A receptor (ADORA2A) had dominant transcript abundance (relative mRNA expression 18.5). Receptor blockade experiments (Figure 3D) further pinpointed the mechanism: the A2AR antagonist ZM241385 robustly blocked and reversed CD206 positivity from 75% to 22%, whereas the A1R antagonist DPCPX did not change the polarization state (CD206+ remained at 73%). These results suggest that A2AR is a major receptor mediating adenosine-driven macrophage polarization in the present *in vitro* system, although contribution from A2BR signaling should be further evaluated in future receptor-specific experiments.

### The adenosine-A2AR axis activates the cAMP/PKA/CREB cascade to drive a pro-tumor transcriptional program

**FIGURE 4:**
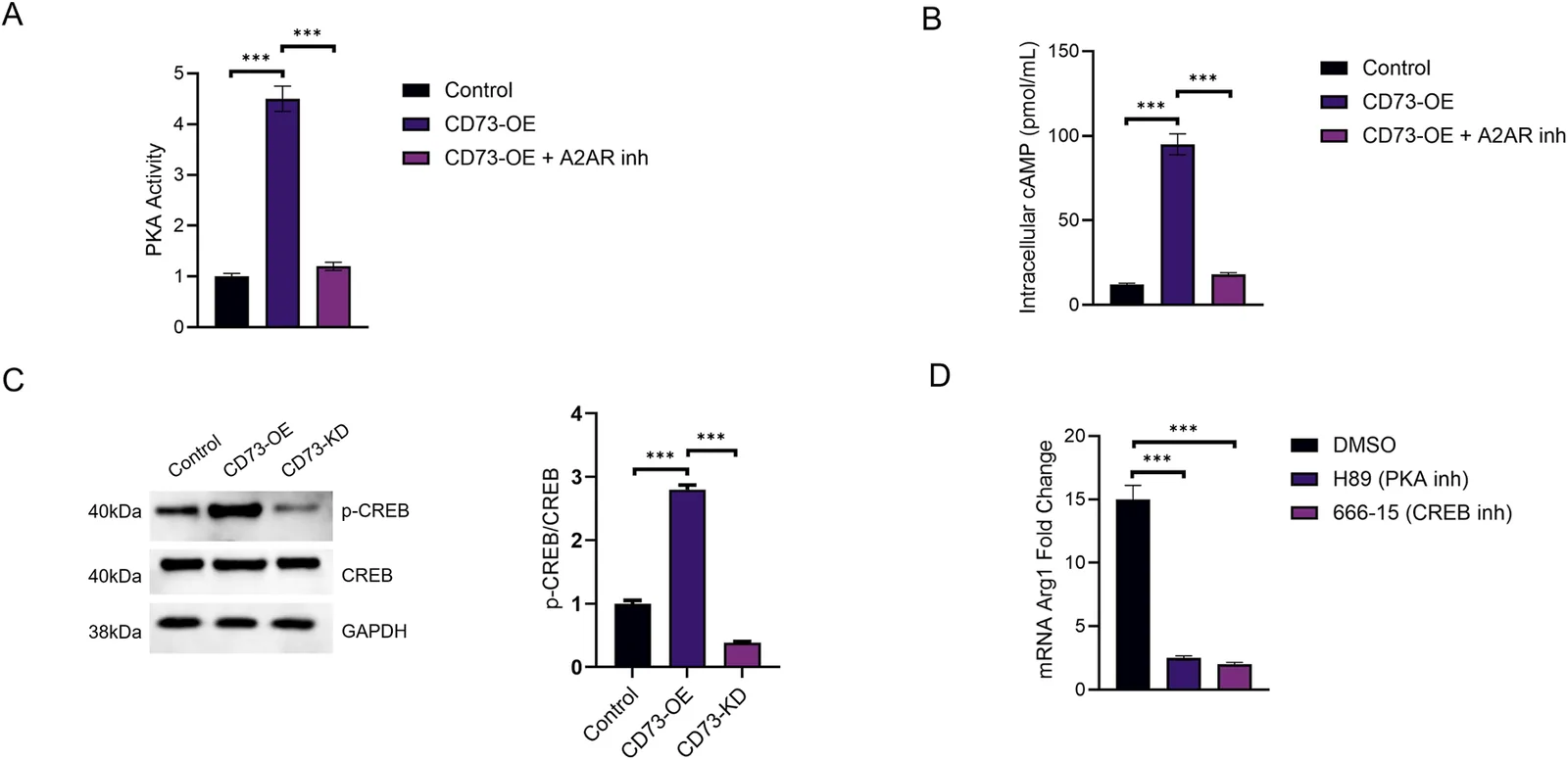
Adenosine-A2AR signaling activates the intracellular cAMP/PKA/CREB cascade. **(A)** ELISA measurement of intracellular cAMP in macrophages under different co-culture conditions and after A2AR antagonist treatment. **(B)** PKA activity units in macrophages across treatment groups. **(C)** Western blot semi-quantification of the p-CREB (Ser133) to total CREB ratio in macrophages. **(D)** Blockade of the M2 marker Arg-1 mRNA expression by PKA inhibitor (H89) or CREB inhibitor (666–15) in the CD73 overexpression system. Statistical significance is indicated by asterisks above the compared groups (*P < 0.05, **P < 0.01, ***P < 0.001).

Because A2AR is a canonical Gs-coupled receptor, its activation elevates intracellular cAMP levels. ELISA measurements of macrophage lysates (Figure 4A) showed that CD73-OE co-culture conditions induced a burst of intracellular cAMP to 95 pmol/mL (baseline control: 12 pmol/mL). Co-treatment with an A2AR antagonist rapidly reduced cAMP levels to 18 pmol/mL cAMP accumulation then triggered strong activation of PKA. Western blot assessment of phosphorylated PKA substrates (Figure 4B) indicated that CD73 overexpression increased PKA activity units to 4.5, which was also reversed by A2AR inhibition to 1.2.

Critically, PKA activation led to robust phosphorylation of its downstream transcription factor CREB at Ser133. Semi-quantitative analysis (Figure 4C) showed that the p-CREB/total CREB ratio increased to 5.2 in the CD73-OE group, whereas it was markedly suppressed to 0.5 in the CD73 knockdown group. In pathway blockade experiments (Figure 4D), adding the PKA inhibitor H89 or the CREB inhibitor 666–15 to the CD73-OE system blocked transcription of M2-associated genes, reducing Arg-1 mRNA from 15 to 2.5 and 2. Together, these data indicate that adenosine signaling requires an intact intracellular cAMP-PKA-CREB cascade to execute macrophage polarization.

### CD73-driven macrophage polarization promotes T-cell dysfunction and is associated with p-CREB-dependent activation of IL-10 transcription

**FIGURE 5:**
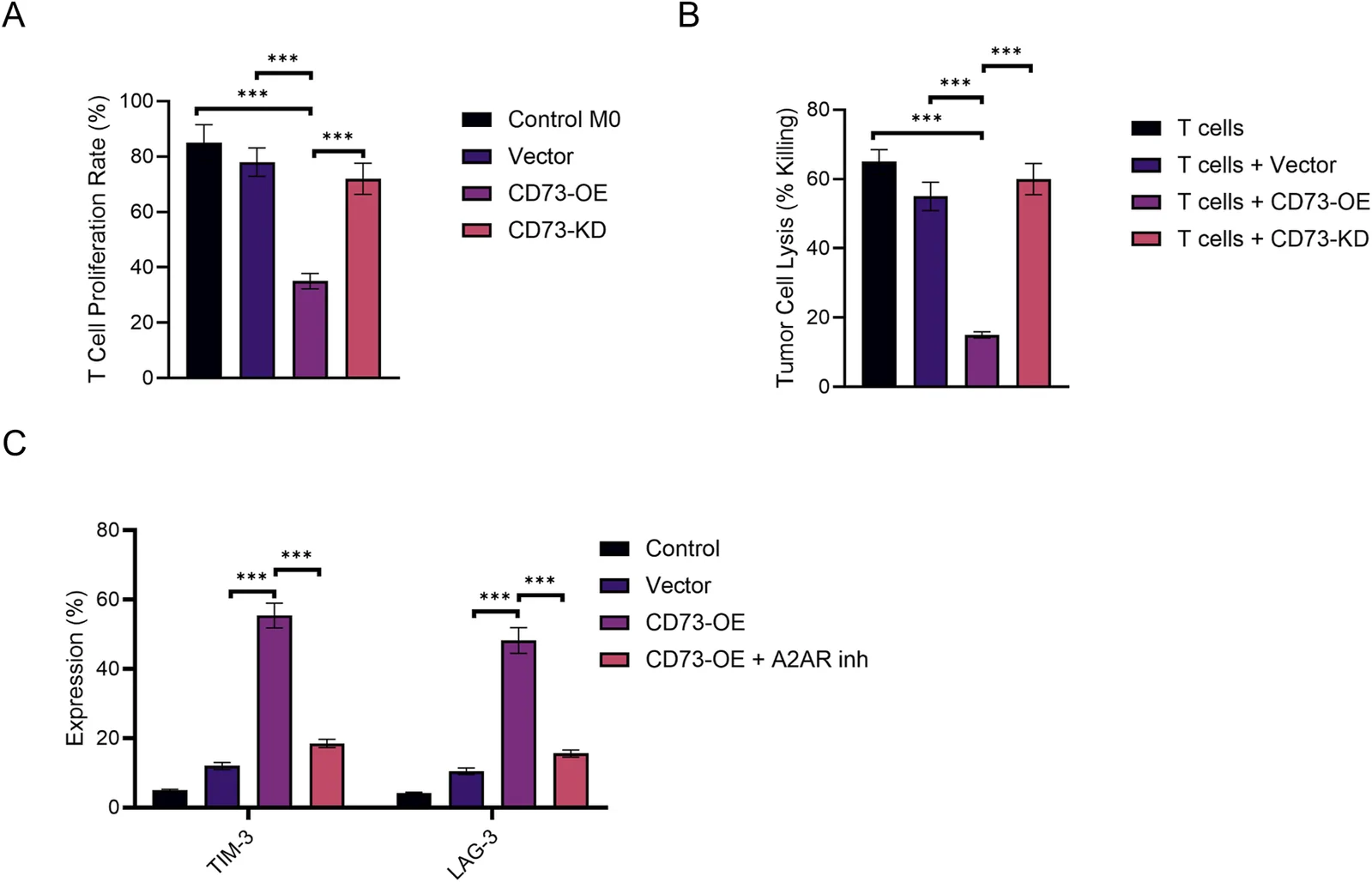
CD73-driven macrophage polarization promotes T-cell dysfunction and exhaustion-associated marker expression. **(A)** Effects of macrophages conditioned under different conditions on proliferation of primary CD8^+^ T cells in the sequential co-culture system. **(B)** Tumor-specific killing rates of T cells exposed to different macrophage paracrine signals. **(C)** Flow cytometry quantification of TIM-3 and LAG-3 expression on T cells and attenuation by upstream A2AR blockade. Statistical significance is indicated by asterisks above the compared groups (*P < 0.05, **P < 0.01, ***P < 0.001).

To evaluate how macrophage reprogramming weakens anti-tumor immune surveillance, a sequential co-culture functional validation system was established. Macrophages conditioned by different tumor cell-conditioned media were co-incubated with activated primary CD8^+^ T cells at a 1:5 ratio. As shown in Figure 5A, T-cell proliferation was suppressed when co-cultured with CD73-OE-conditioned macrophages, with a proliferation rate of 35%, whereas T cells cultured with control (M0-derived) and vector-conditioned macrophages maintained proliferation rates of 85% and 78%, respectively. Cytotoxicity assays further supported this finding (Figure 5B): tumor-specific killing by T cells exposed to CD73-OE macrophage paracrine signals was impaired from 65% (T cells alone) to 15%. Flow cytometry analysis of exhaustion-associated markers (TIM-3 and LAG-3) (Figure 5C) revealed that CD73-OE-conditioned microenvironments increased TIM-3 and LAG-3 positivity to 55.4% and 48.2%, respectively. Notably, introducing an A2AR antagonist during the upstream co-culture attenuated this T-cell dysfunction-associated phenotype, reducing TIM-3 and LAG-3 positivity to 18.5% and 15.6%, respectively.

### p-CREB directly binds and activates IL-10 transcription

**FIGURE 6:**
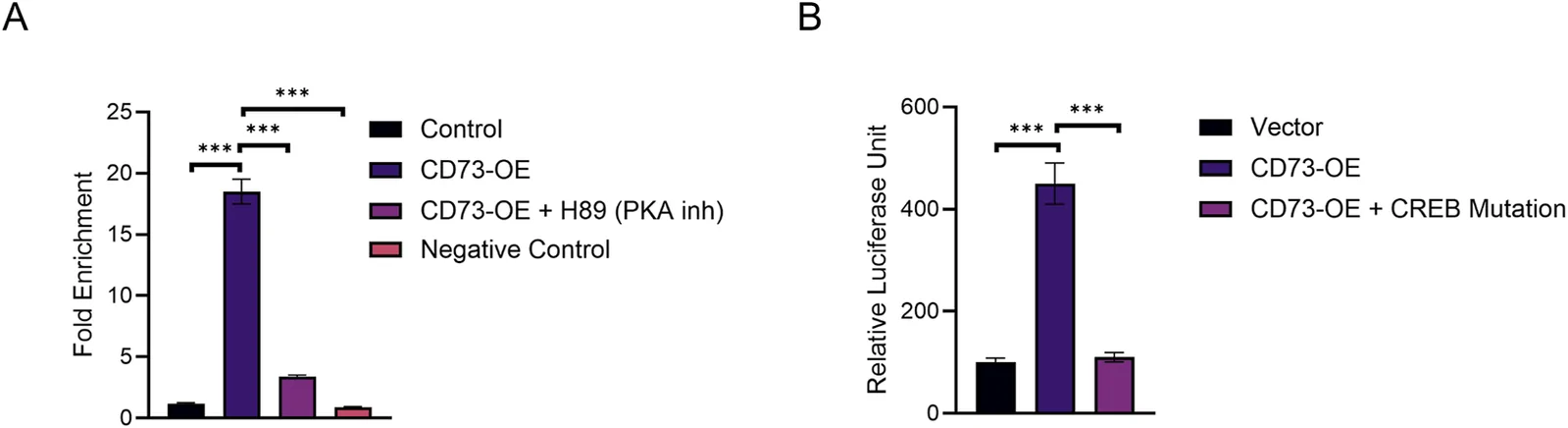
p-CREB directly binds and activates IL-10 transcription. **(A)** ChIP-qPCR validation of p-CREB binding to the IL-10 promoter and corresponding DNA enrichment under different treatments. **(B)** Dual-luciferase reporter assay comparing transcriptional activity of the wild-type IL-10 promoter versus a CREB-binding-motif mutant after stimulation with CD73-OE conditioned media (RLU). Statistical significance is indicated by asterisks above the compared groups (*P < 0.05, **P < 0.01, ***P < 0.001).

Finally, we investigated how CREB confers macrophages with a paracrine program that suppresses T-cell function at the promoter level. ChIP-qPCR assays (Figure 6A) demonstrated that, under CD73 overexpression conditions, p-CREB specifically bound the IL-10 promoter with DNA enrichment up to 18.5-fold (compared with 1.2-fold in controls). Blocking upstream signaling with the PKA inhibitor H89 substantially reduced this enrichment (to 3.4-fold). Dual-luciferase reporter assays (Figure 6B) provided direct support: luciferase reporters carrying the wild-type IL-10 promoter showed increased transcriptional activity to 450 RLU after stimulation with CD73-OE conditioned media. In contrast, mutating the CREB-binding motif in the promoter sharply reduced activity to a baseline level of 110 RLU. This molecular interaction supports p-CREB as an important downstream effector linking adenosine signaling to IL-10 transcription and immunosuppressive cytokine production in this *in vitro* NSCLC model.

## Discussion

The present study links tumor-cell CD73 expression with an immunosuppressive macrophage phenotype in NSCLC. TCGA-based analyses suggested that high CD73 (NT5E) expression was associated with poorer overall survival and positively correlated with the M2 macrophage marker CD206/MRC1. Although these clinical and database analyses are correlative, they provide a rationale for experimentally testing whether CD73-dependent purine metabolism contributes to macrophage reprogramming in the tumor microenvironment. Prior NSCLC tissue analyses have likewise identified CD73 and A2AR as clinically relevant prognostic markers, with high CD73 expression associated with worse outcomes (Inoue et al., 2017). In EGFR-mutated NSCLC models, combined anti-PD-L1 and anti-CD73 therapy has been reported to enhance T-cell responses, supporting the translational relevance of targeting this axis (Tu et al., 2022).

Functional experiments supported this mechanism. CD73 overexpression in A549 cells markedly increased extracellular adenosine production, whereas CD73 knockdown in H1299 cells reduced adenosine output. In the Transwell co-culture system, CD73-high tumor cells promoted an M2-like macrophage phenotype, as indicated by elongated morphology, increased CD206 expression, elevated IL-10/TGF-β secretion, and reduced CD86/TNF-α. Rescue with exogenous adenosine or NECA restored M2 polarization under CD73-deficient conditions, while A2AR blockade reversed the CD73-driven phenotype. These findings identify adenosine-A2AR signaling as a major mediator of tumor-cell CD73-associated macrophage polarization *in vitro*, while leaving open the possibility that A2BR or other adenosine receptor pathways may contribute under different microenvironmental conditions. A2AR-dependent enhancement of IL-10 production in macrophages has also been demonstrated in other experimental contexts, supporting the biological plausibility of the adenosine-A2AR-IL-10 link observed here (Csoka et al., 2007).

Downstream signaling analysis further clarified the intracellular pathway. Activation of A2AR increased macrophage cAMP levels, enhanced PKA activity, and promoted CREB phosphorylation at Ser133. Pharmacological inhibition of PKA or CREB markedly reduced Arg-1 expression, indicating that the cAMP/PKA/CREB cascade is required for execution of the M2-associated transcriptional program. ChIP-qPCR and dual-luciferase reporter assays further showed that phosphorylated CREB can bind and activate the IL-10 promoter, providing a direct transcriptional link between adenosine sensing and immunosuppressive cytokine production.

The sequential co-culture experiments suggest that CD73-conditioned macrophages can impair CD8^+^ T-cell immunity. Macrophages exposed to CD73-overexpressing tumor cells reduced T-cell proliferation and tumor-cell killing while increasing the exhaustion-associated markers TIM-3 and LAG-3. Because TIM-3 and LAG-3 alone do not fully define terminal T-cell exhaustion, these findings are best interpreted as evidence of T-cell dysfunction with an exhaustion-associated phenotype. Importantly, upstream A2AR blockade attenuated these effects, supporting a pathway in which tumor-cell CD73 generates adenosine, adenosine activates macrophage A2AR-cAMP/PKA/CREB signaling, and the resulting M2-like paracrine program contributes to T-cell dysfunction. This interpretation is consistent with the broader concept that adenosine-rich tumor microenvironments suppress anti-tumor immunity, but the present conclusions should be understood in the context of the experimental limitations described below. This concept is consistent with the established hypoxia-A2-adenosinergic model of tumor immune suppression (Sitkovsky et al., 2014). IL-10 is also a broadly expressed immunoregulatory cytokine whose production is controlled through multiple transcriptional and post-transcriptional mechanisms across immune-cell types (Saraiva and O’Garra, 2010).

### Study limitations

Several limitations should be acknowledged. First, the mechanistic experiments were performed mainly *in vitro* using NSCLC cell lines, THP-1-derived macrophages, and primary CD8^+^ T cells; therefore, the findings require validation in in vivo models, such as syngeneic or orthotopic immunocompetent models and, where human immune-cell interactions are specifically examined, humanized patient-derived xenograft models. Second, TCGA-based and clinical-sample correlation analyses cannot establish causality. Third, although A2AR blockade produced a strong reversal effect, the potential contribution of A2BR signaling was not fully excluded and should be examined using receptor-specific genetic and pharmacological approaches. Fourth, donor-to-donor heterogeneity of primary CD8^+^ T cells may influence functional readouts. Future studies should integrate *in vivo* validation, spatial profiling of patient tissues, and receptor-specific genetic interventions, especially A2AR and A2BR gain- and loss-of-function approaches, to further confirm the translational relevance of the CD73-adenosine axis in NSCLC.

## Conclusions

In summary, this study delineates the core molecular network of the CD73-adenosine-A2AR-cAMP/PKA/CREB signaling axis in driving M2-like polarization of macrophages in NSCLC-related *in vitro* models. These findings provide mechanistic evidence for a metabolically driven immunosuppressive network in the tumor immune microenvironment and support further preclinical validation of immunometabolic checkpoint strategies targeting CD73 or adenosine receptors, particularly A2AR and potentially A2BR, to reverse macrophage-mediated T-cell dysfunction and improve immunotherapy responses in NSCLC.

## Data Availability

The original contributions presented in the study are included in the article/supplementary material, further inquiries can be directed to the corresponding author.
